# Future directions for monitoring and human health research for the Arctic Monitoring and Assessment Programme

**DOI:** 10.1080/16549716.2018.1480084

**Published:** 2018-06-26

**Authors:** B. Adlard, S. G. Donaldson, J.O. Odland, P. Weihe, J. Berner, A. Carlsen, E. C. Bonefeld-Jorgensen, A. A. Dudarev, J.C. Gibson, E.M. Krümmel, K. Olafsdottir, K. Abass, A. Rautio, I.A. Bergdahl, G. Mulvad

**Affiliations:** a Health Canada, Ottawa, Canada; b Department of Community Medicine (UiT), The Arctic University of Norway, Tromso, Norway; c Department of Occupational Medicine and Public Health, The Faroese Hospital System, Torshavn, Faroe Islands; d Alaska Native Tribal Health Consortium, Anchorage, AK, USA; e Department of Public Health, Aarhus University, Aarhus, Denmark; f Center for Arctic Health, Department of Public Health, Aarhus University, Aarhus, Denmark; g Greenland Center for Health Research, Institute of Nursing and Health Science, University of Greenland, Nuuk, Greenland; h Northwest Public Health Research Center, St. Petersburg, Russia; i Inuit Circumpolar Council, Ottawa, Canada; j Department of Pharmacology and Toxicology, University of Iceland, Reykjavik, Iceland; k Faculty of Medicine, Arctic Health, University of Oulu, Oulu, Finland; l Thule Institute and Faculty of Medicine, University of Oulu, Oulu, Finland; m Occupational and Environmental Medicine, Department of Public Health and Clinical Medicine, Umeå University, Sweden

**Keywords:** Arctic, human health, biomonitoring, environmental contaminants

## Abstract

For the last two and a half decades, a network of human health experts under the Arctic Monitoring and Assessment Program (AMAP) has produced several human health assessment reports. These reports have provided a base of scientific knowledge regarding environmental contaminants and their impact on human health in the Arctic. These reports provide scientific information and policy-relevant recommendations to Arctic governments. They also support international agreements such as the Stockholm Convention on Persistent Organic Pollutants (POPs) and the Minamata Convention on Mercury. Key topics discussed in this paper regarding future human health research in the circumpolar Arctic are continued contaminant biomonitoring, health effects research and risk communication. The objective of this paper is to describe knowledge gaps and future priorities for these fields.

## Background

In 1991, the Arctic Monitoring and Assessment Programme (AMAP) was established with a mandate to develop a monitoring programme targeting Arctic pollutants. With the establishment of Arctic Council in 1996, AMAP became a working group of the Arctic Council. In more recent years, this AMAP monitoring work has also considered the impacts of environmental stressors such as climate change. The Human Health Assessment Group (HHAG) is an expert group under AMAP that assesses contaminant levels in human populations of the Arctic and contaminant impacts on human health. Arctic monitoring and research is important for understanding contaminant trends and health risks, but also providing insight into the effectiveness of public health strategies, policies, and international mitigation actions to reduce exposure to contaminants. The AMAP HHAG coordinates and creates opportunities for monitoring and research across the circumpolar Arctic, and has published several human health assessment reports [1–4]. These reports have provided reliable and up-to-date information which has formed the scientific basis for policy recommendations, and has ensured that public health decisions are based on the best available knowledge.

These AMAP human health assessments contribute to important international conventions such as the:

- United Nations Economic Commission for Europe Convention on Long-Range Transboundary Air Pollution protocols on Persistent Organic Pollutants (POPs) and metals
- United Nations Stockholm Convention on POPs
- United Nations Minamata Convention on Mercury

The latest human health report from 2015 was a comprehensive compilation of current knowledge regarding the presence of contaminants in human populations, health effects and risks, risk communication and issues related to adaptation to environmental changes [1].

## Knowledge gaps and future priorities

Despite many challenges associated with conducting research and monitoring programs in the Arctic [5], the results have been valuable for Indigenous communities, policy makers and public health officials. Many lessons have been learned including the value of culturally appropriate risk communications while addressing what is often referred to as the ‘Arctic dilemma’: balancing the benefits of harvesting and consuming traditional foods (often referred to as country foods or food from subsistence activities such as hunting, gathering, trapping and fishing) with the potential health risks due to the dietary intake of environmental contaminants.

The latest human health report describes a number of knowledge gaps that have yet to be addressed [1]. In response, the AMAP HHAG has developed a strategic plan outlining future priorities and opportunities based on key recommendations from the AMAP report and input from international Arctic subject matter experts, who participated in AMAP HHAG expert group discussions.

## Methods

The AMAP HHAG holds meetings on a biannual basis. Over the course of two meetings held in 2015, the strategic plan was opened for discussion and input by participating AMAP HHAG members, including Key National Experts and Designated Experts from all eight circumpolar countries, and Permanent Participant organizations of the Arctic Council. This discussion formed the basis and general framework for this manuscript, which was initially proposed at a 2016 meeting. A draft manuscript was presented to the AMAP HHAG for further input, review, and discussion at two AMAP HHAG meetings in 2017. This manuscript outlines the key themes for future work as highlighted by the AMAP HHAG, which are categorized under the following headings: monitoring, health effects research, and risk communication.

## Monitoring

As the main route of exposure to POPs and metals in the Arctic has historically been consumption of traditional foods, there remains a need for the coordinated collection of population dietary information. Further research and new methods/approaches are needed to accurately estimate food consumption, including variation in consumption patterns due to seasonality. Special consideration should be given to understanding how climate change may influence availability and access to food, as well as factors that may influence food safety (e.g. changes in the bioavailability and movement of contaminants, contaminant-related diseases in wildlife, and the prevalence of pathogens or parasites). Further investigation of other potential sources of contaminant exposure in the Arctic is also needed.

A crucial part of the core AMAP monitoring programme has been continuous long term maternal biomonitoring. Maternal biomonitoring of POPs and metals, and mother/child cohort studies have provided invaluable information about exposure to contaminants during critical stages of development (fetal and childhood exposure). These studies will remain a priority in the future; however, future studies should also consider adult male exposure, possibly through studies of fathers in parents/child cohorts.

### ‘New and emerging’ contaminants

Limited human biomonitoring and health effects data for several POPs newly listed or being proposed for inclusion on the Stockholm Convention (e.g. dicofol, and perfluoroalkyl acids [PFAAs] such as perfluorooctanoic acid [PFOA]), were identified as knowledge gaps in the AMAP 2015 report. Future biomonitoring should aim to track trends in contaminants levels, identify chemicals of emerging concern, and provide baseline data for new POPs as analytical methods for human samples become available.

### Quality of analysis

A key aspect of the core AMAP monitoring programme is the continued strengthening and participation in the AMAP Ring Test. The AMAP Ring Test ensures that contaminants data from all participating labs are of high quality and comparability, and currently includes 32 laboratories and 37 different analytes. This is critical for demonstrating that all data used in AMAP reports, such as spatial and temporal trends across the Arctic, can be reported with confidence and accuracy.

### Cross-disciplinary efforts and benefits

Due to the many emerging challenges and health concerns in the Arctic, future studies should be designed in a collaborative nature to create cross-disciplinary studies that will address multiples issues. This will result in more efficient use of time, reduce cost and resources, and will produce studies that can collect information on multiple key issues that require further research including: contaminants (including sources of exposure), zoonotic diseases, circumpolar food and water security, impact of climate change, and potential interactions between these issues. These studies should also encompass the harmful effects from the loss of knowledge transfer and rapid food transition from traditional subsistence hunting and gathering to expensive imported market foods in some Arctic regions. AMAP relies on each of the Arctic countries’ National Implementation Plans in order to produce the research and monitoring data needed to address Arctic concerns. In the future, the AMAP HHAG will continue to coordinate Arctic research efforts and work with experts from other Arctic Council working groups, as well as academics, governmental agencies, and Indigenous Peoples/Permanent Participant organizations’ representatives to identify opportunities to address mutual priorities.

## Health effects

The AMAP Human Health programme has assessed and documented possible health effects, due to exposure to priority contaminants, primarily through epidemiological studies such as mother/child cohort studies [6]. The continued development of representative, prospective cohort studies is a priority, as it is an efficient way to obtain information on health risks from contaminants in relation to nutrition, lifestyle, climate change and genetic factors.

### Core health effects programme

Recommendations and priorities for the AMAP Human Health programme include continued/expanded research into health effects such as: immunological, neurobehavioral, cardiovascular, metabolic, diabetogenic, developmental, reproductive, endocrine, and epigenetic. Future research studies should aim to investigate potential chemical interactions and combined effects on health outcomes. These studies also need to consider possible interactions between contaminants and dietary nutrients which may mitigate observed health outcomes.

### Harmonization and data comparability

Conducting studies in the Arctic is challenging and expensive, and while studies should be tailored to address local issues, there needs to be a harmonization of study designs and reporting of results to allow for the possibility of merging studies and performing strong meta-analyses [7]. This will enhance the value of the data and allow for better comparisons with other regions including those outside of the Arctic.

### Coordination and collaboration

Health studies should be developed with relevant public health authorities to investigate general public health issues, and potential impacts of climate change and lifestyle changes. Research studies that investigate the availability and quality of traditional foods, and the potential impact from climate change or contaminant-related diseases in traditional wildlife species, should work in close collaboration with other expert groups under the Arctic Council. The development of joint cohorts and research projects will support achieving multiple objectives, bridging various research initiatives, and reducing the burden of repeated visits to participating local communities, which can cause research fatigue.

## Risk communication

Risk assessment, the evaluation of all the available scientific evidence resulting in a determination of ‘safe levels’ of exposure, is the foundation for risk communication. Description of risk varies across jurisdictions. Risk communication and providing health messaging related to contaminants is a complex undertaking in the Arctic, as there are a number of important issues to consider including: socio-economic, cultural, spiritual, environmental, dietary, and overall food security [8]. There is limited research available that assesses the effectiveness of risk communication messages.

Future studies in risk communication effectiveness should consider other influencing local factors such as risk perceptions, food security, health priorities, dietary patterns, determinants of food choice, and local or regional environmental issues, which may vary across Arctic regions.

## Conclusion

AMAP has an ongoing mandate to provide the most current and best available information on issues regarding environmental contaminants and human health in the Arctic. By coordinating Arctic biomonitoring to provide comparable datasets, preparing comprehensive assessments, and providing recommendations to the scientific community to guide future Arctic research, the AMAP HHAG is able to provide relevant information for international agreements and conventions such as the United Nations Stockholm and Minamata Conventions.

The work of the AMAP HHAG supports the overall AMAP strategy, which enables AMAP to continue to:

- address existing knowledge gaps and emerging Arctic concerns
- produce high quality scientific reports that include the best available knowledge
- provide policy-relevant and science-based advice to health officials, policy makers and Arctic governments
- make knowledge available and usable for local communities, and facilitate open dialogue regarding environmental and health care issues.

## Paper context

Contaminants in the Arctic represent a potential human health concern, particularly populations that regularly consume Arctic wildlife. Due to the changing Arctic environment, the human health impact from contaminants, zoonotic diseases, food/water security, climate change and their potential interactions, remains relatively unknown. The Arctic Monitoring and Assessment Programme have produced several reports which summarize current knowledge. This short communication provides recommendations for future research directions in the Arctic to identify priorities and support cross-disciplinary collaborations.
